# Supplementary material for: Robust bilayer solid electrolyte interphase for Zn electrode with high utilization and efficiency
Source: Nat Commun. 2024 Sep 29;15:8431. doi: 10.1038/s41467-024-52611-z (PMC11439932; doi:10.1038/s41467-024-52611-z)
Supplement: Supplementary file 1 — Supplementary Information [file 41467_2024_52611_MOESM1_ESM.pdf]

Supplementary Information

Robust Bilayer Solid Electrolyte Interphase for Zn Electrode with High Utilization and Efficiency

Yahan Meng[1,†], Mingming Wang[1,†], Jiazhi Wang[2,†], Xuehai Huang[3], Xiang Zhou[3], Muhammad Sajid[1], Zehui Xie[1], Ruihao Luo[1], Zhengxin Zhu[1], Zuodong Zhang[1], Nawab Ali Khan[1], , Yu Wang[3,], Zhenyu Li[2,*], Wei Chen[1,*]*

[1] Department of Applied Chemistry, School of Chemistry and Materials Science, Hefei National Research Center for Physical Sciences at the Microscale, University of Science and Technology of China, Hefei, Anhui 230026, China
[2] Key Laboratory of Precision and Intelligent Chemistry, University of Science and Technology of China, Hefei, Anhui 230026, China
[3] Center for Electron Microscopy, South China Advanced Institute for Soft Matter and Guangdong Provincial Key Laboratory of Functional and Intelligent Hybrid Materials and Devices, School of Emergent Soft Matter, South China University of Technology, Guangzhou 510640, China.

[†] Y.M., M.W. and J. W. contributed equally to this work.

[*]*Corresponding authors:* weichen1@ustc.edu.cn
zyli@ustc.edu.cn
roywangyu@scut.edu.cn

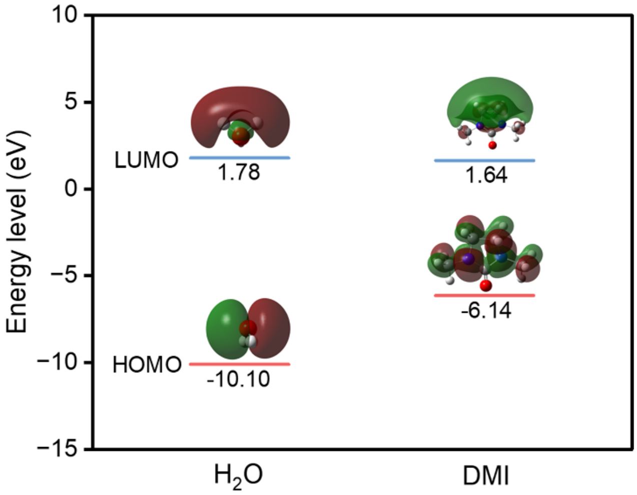

Supplementary Fig. 1. HOMO/LUMO energy levels of H_2O and DMI molecules.

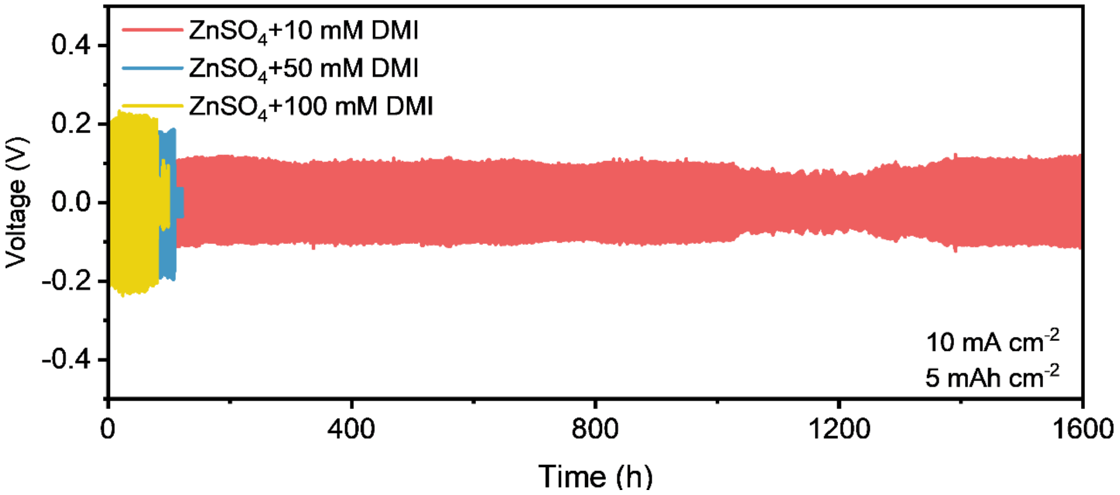

Supplementary Fig. 2. Long-term stability of Zn||Zn symmetric cells in 2 M $ZnSO_4$ electrolytes with different concentrations of DMI at 5 mAh cm^{-2} and 10 mA cm^{-2} (25 °C). With the increase in DMI concentration, the cycling life of the battery significantly decreases, and the overpotential significantly increases. This may be due to (1) the decrease in the ionic conductivity of the electrolyte (as shown in Supplementary Fig. 3) and (2) the excessively thick SEI formed by the high concentration of DMI decomposition, leading to difficult and uneven Zn^{2+} transport.

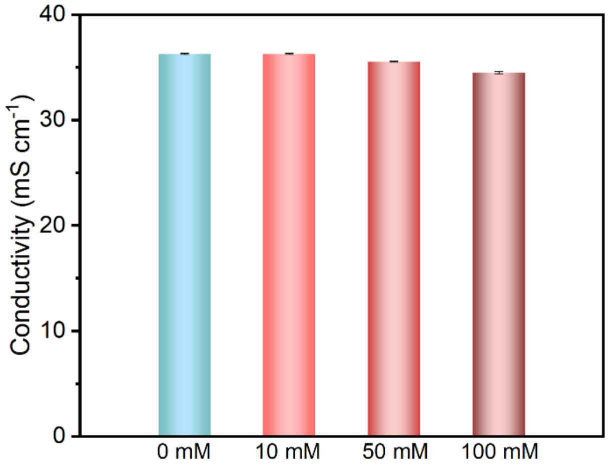

Supplementary Fig. 3. The ionic conductivity of 2 M $ZnSO_4$ electrolytes with different DMI concentrations (25 ℃). The meaning of error bar is the instrumental error in three tests of each electrolyte.

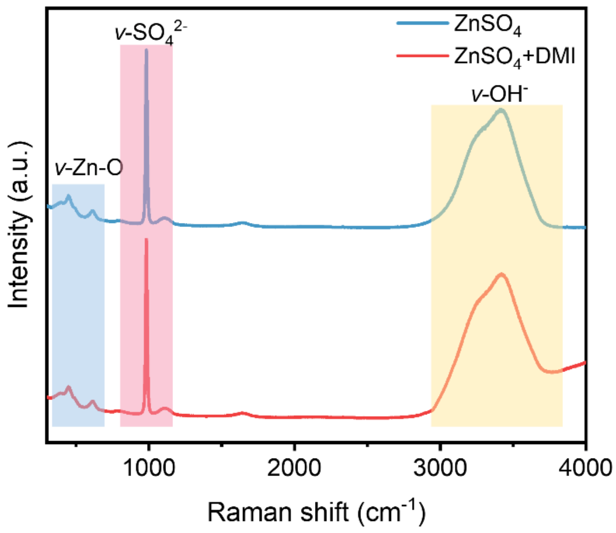

Supplementary Fig. 4. The Raman spectra of 2 M $ZnSO_4$ and 2 M $ZnSO_4$ + 10 mM DMI electrolytes.

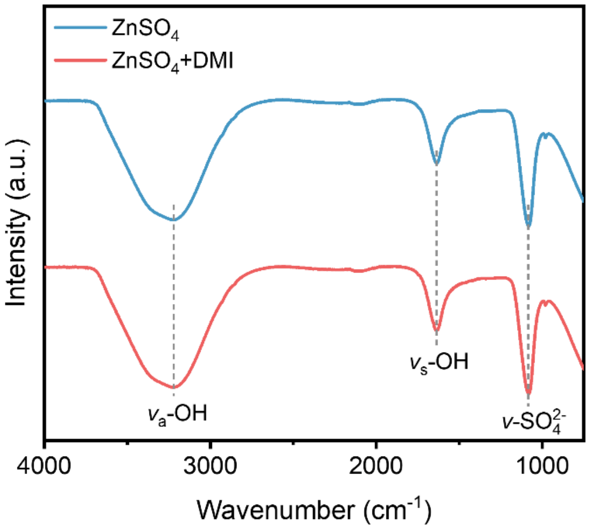

Supplementary Fig. 5. The FT-IR spectra of 2 M $ZnSO_4$ and 2 M $ZnSO_4$ + 10 mM DMI electrolytes.

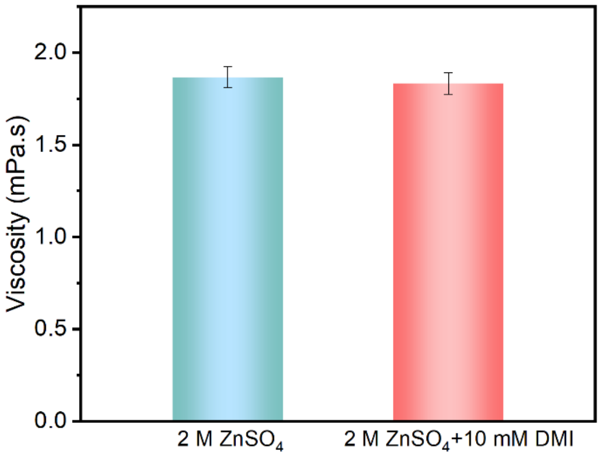

Supplementary Fig. 6. The viscosity of 2 M $ZnSO_4$ and 2 M $ZnSO_4$ + 10 mM DMI electrolytes (25 ℃). The meaning of error bar is the instrumental error in three tests of each electrolyte.

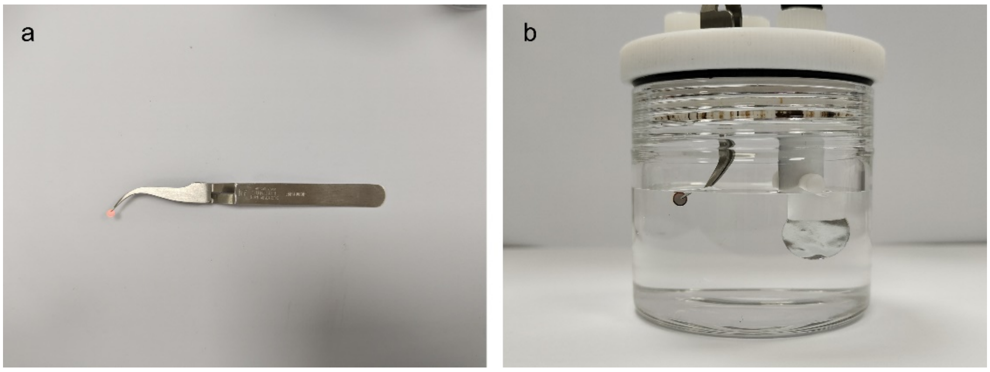

Supplementary Fig. 7. Digital photographs of the in-situ TEM setup for Zn electrodeposition.

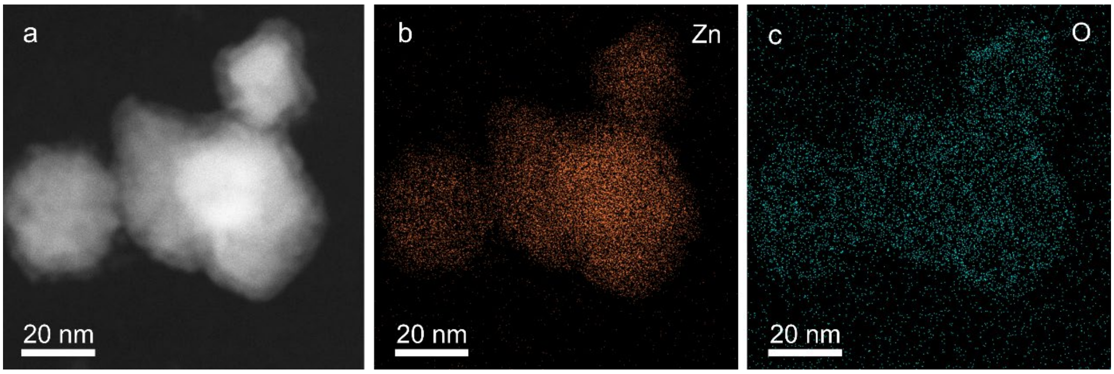

Supplementary Fig. 8. a, STEM image of Zn deposition in $ZnSO_4$ electrolyte without SEI. **b-c**, The corresponding EDS mapping of (**a**).

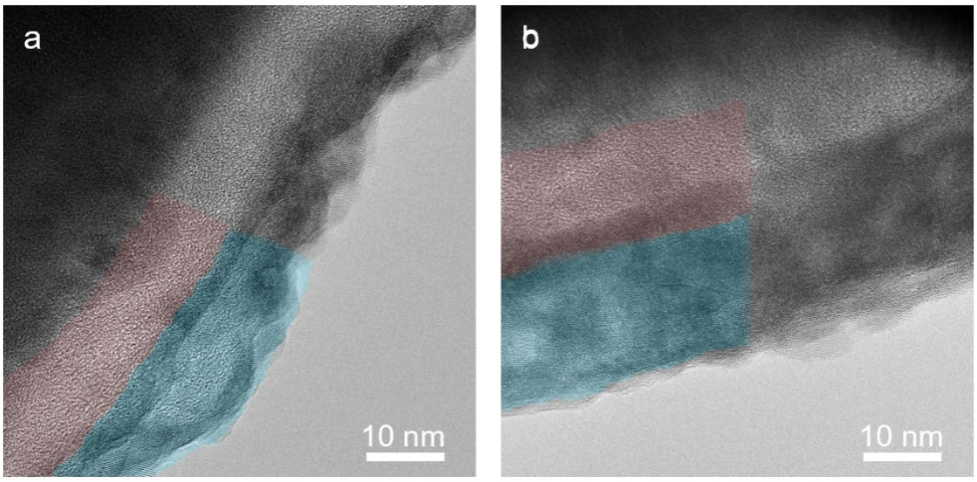

Supplementary Fig. 9. TEM images of bilayer SEI on Zn surface in different regions in $ZnSO_4$-DMI electrolyte (The light pink area represents the amorphous inner layer of the bilayer SEI and the light blue area is the crystalline outer layer of the bilayer SEI.).

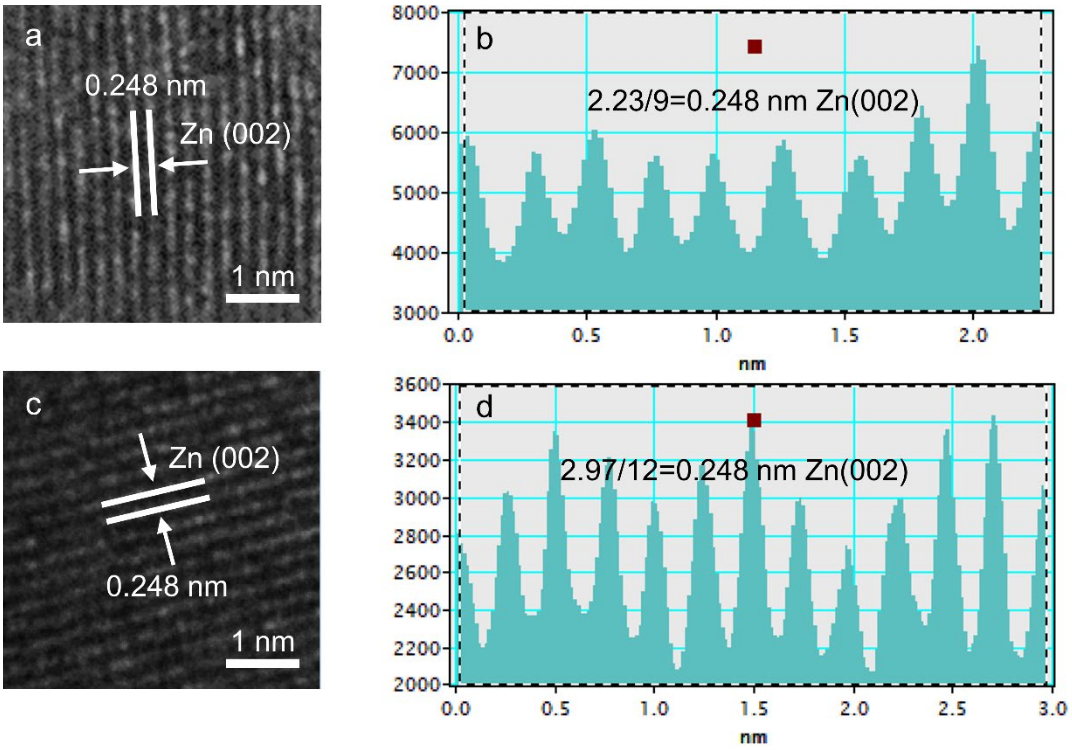

Supplementary Fig. 10. a and **c**, HRTEM images of Zn depositions. **b** and **d**, The intensity profiles of the lattice parameters of Zn deposition.

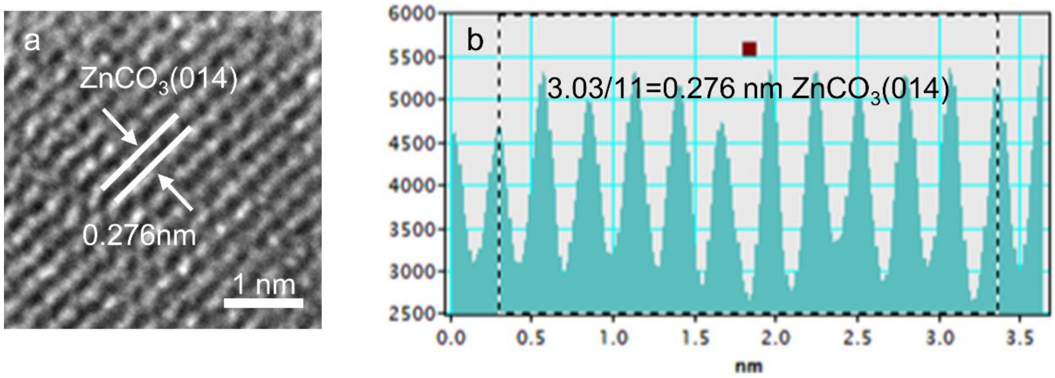

Supplementary Fig. 11. a, HRTEM images of $ZnCO_3$ in the outer layer of the bilayer SEI. **b**, The intensity profiles of the lattice parameters of $ZnCO_3$ in the outer layer of the bilayer SEI.

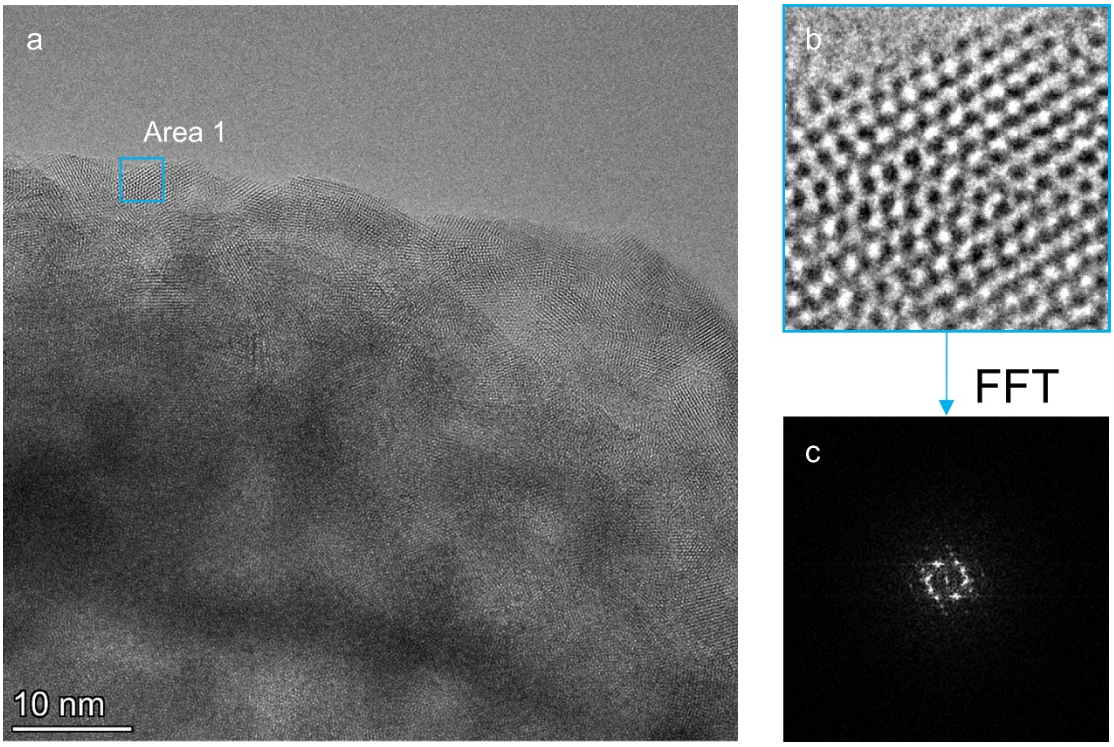

Supplementary Fig. 12. **a**, Aberration-corrected TEM image of crystalline area in the outer layer of the bilayer SEI. **b**, HRTEM image of area 1 labeled in Supplementary Fig. 12a. **c**, Fast Fourier transform corresponding to (**b**).

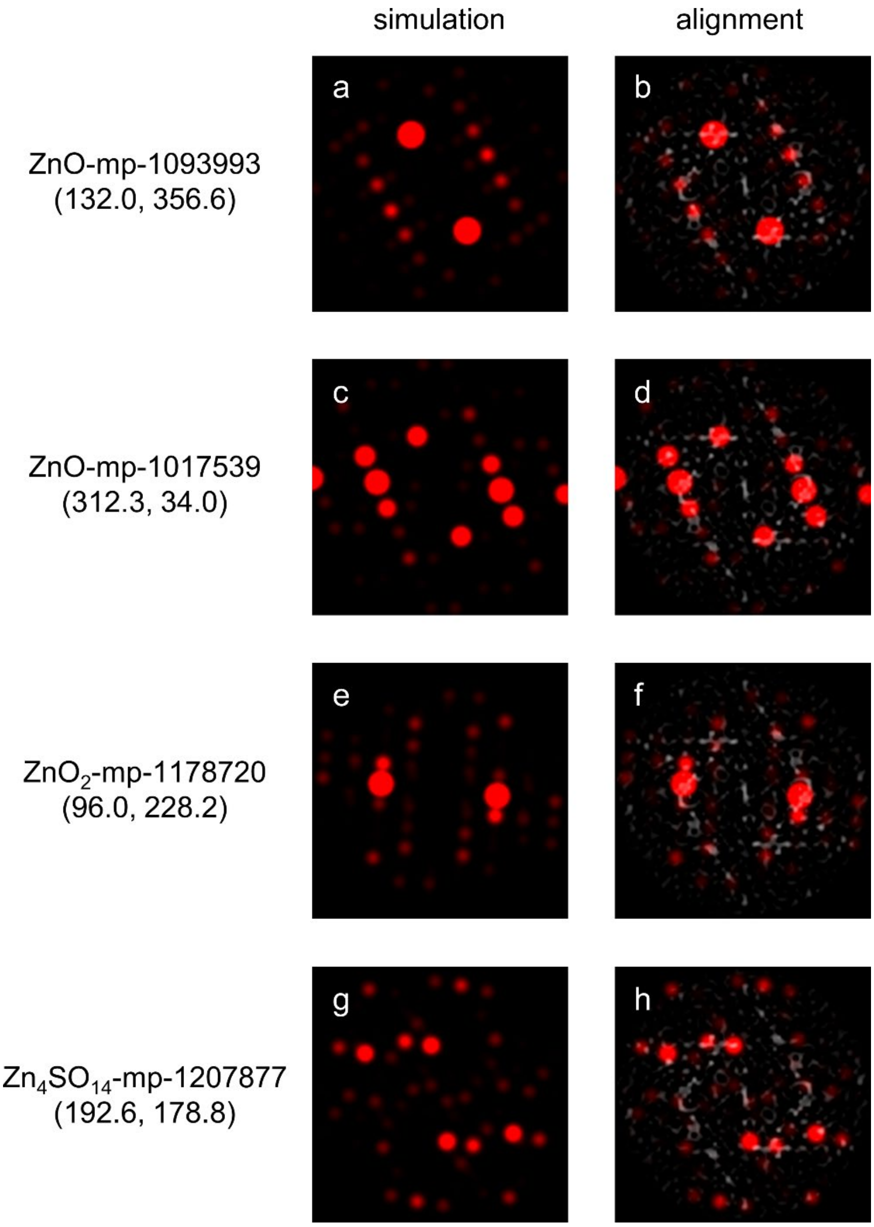

Supplementary Fig. 13. Theoretical reciprocal space of (**a**) ZnO-mp-1093993, (**c**) ZnO-mp-1017539, (**e**) ZnO_2-mp-1178720 and (**g**) Zn_4SO_{14}-mp-1207877. (**b, d, f, h**) The alignment of theoretical vs. actual reciprocal space corresponding to area 1 labeled in Supplementary Fig. 12.

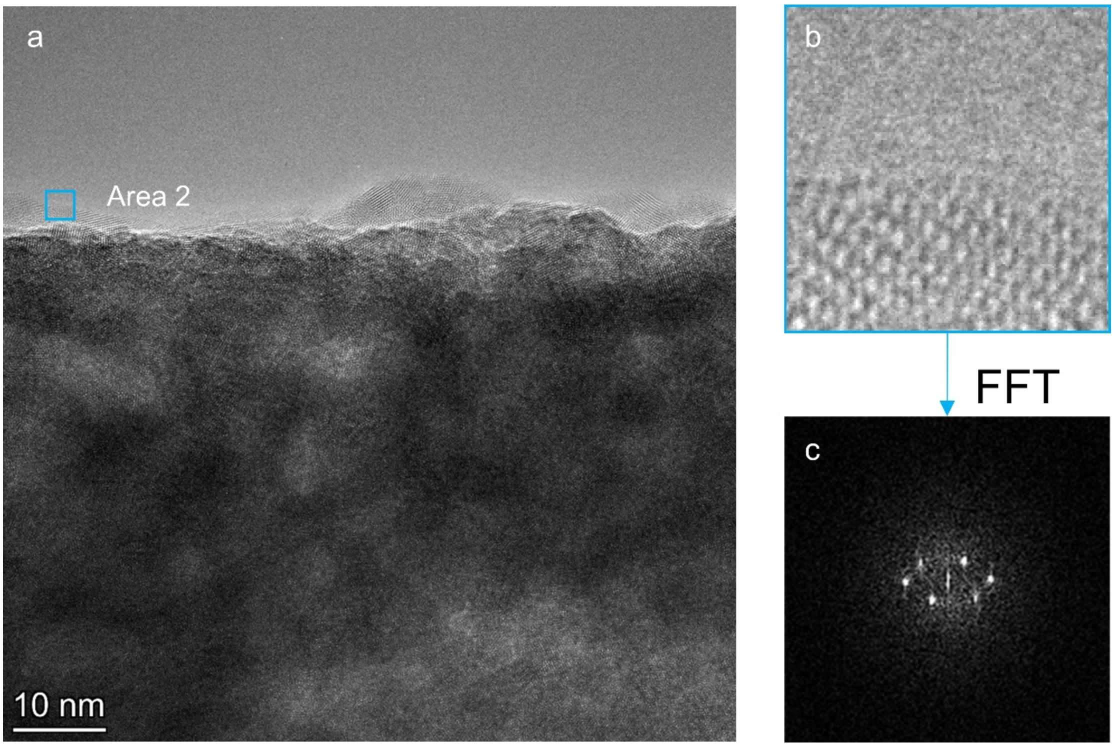

Supplementary Fig. 14. a, Aberration-corrected TEM image of the crystalline area in the outer layer of the bilayer SEI. **b**, HRTEM image of area 2 labeled in Supplementary Fig. 14a. **c**, Fast Fourier transform corresponding to (**b**).

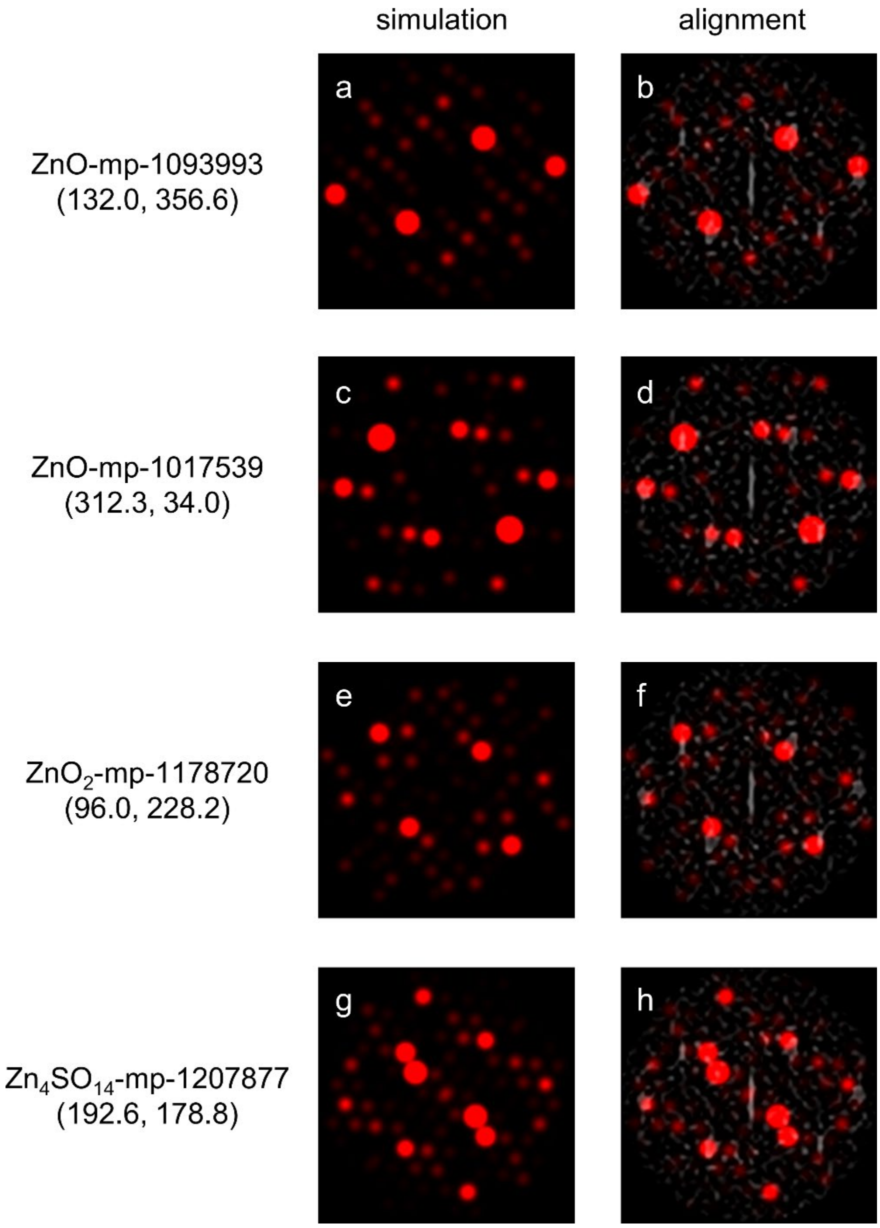

Supplementary Fig. 15. Theoretical reciprocal space of (**a**) ZnO-mp-1093993, (**c**) ZnO-mp-1017539, (**e**) ZnO_2-mp-1178720 and (**g**) Zn_4SO_{14}-mp-1207877. (**b, d, f, h**) The alignment of theoretical vs. actual reciprocal space corresponding to area 2 labeled in Supplementary Fig. 14.

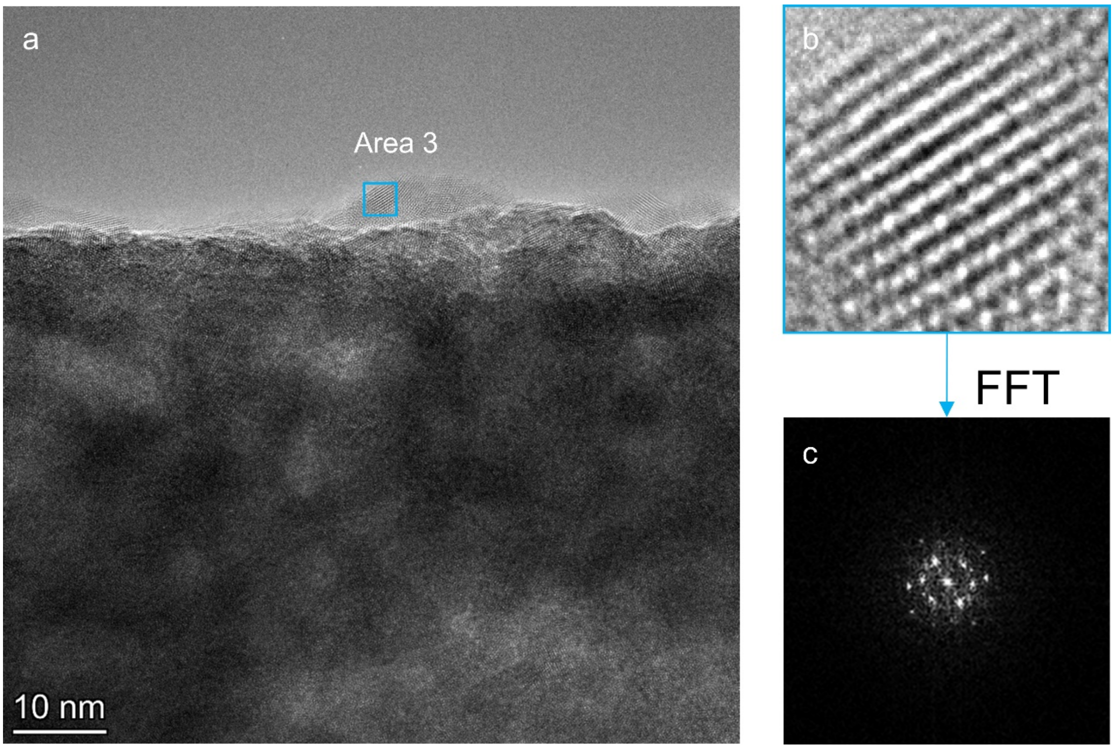

Supplementary Fig. 16. a, Aberration-corrected TEM image of crystalline area in the outer layer of the bilayer SEI. **b**, HRTEM image of area 3 labeled in Supplementary Fig. 16a. **c**, Fast Fourier transform corresponding to (**b**).

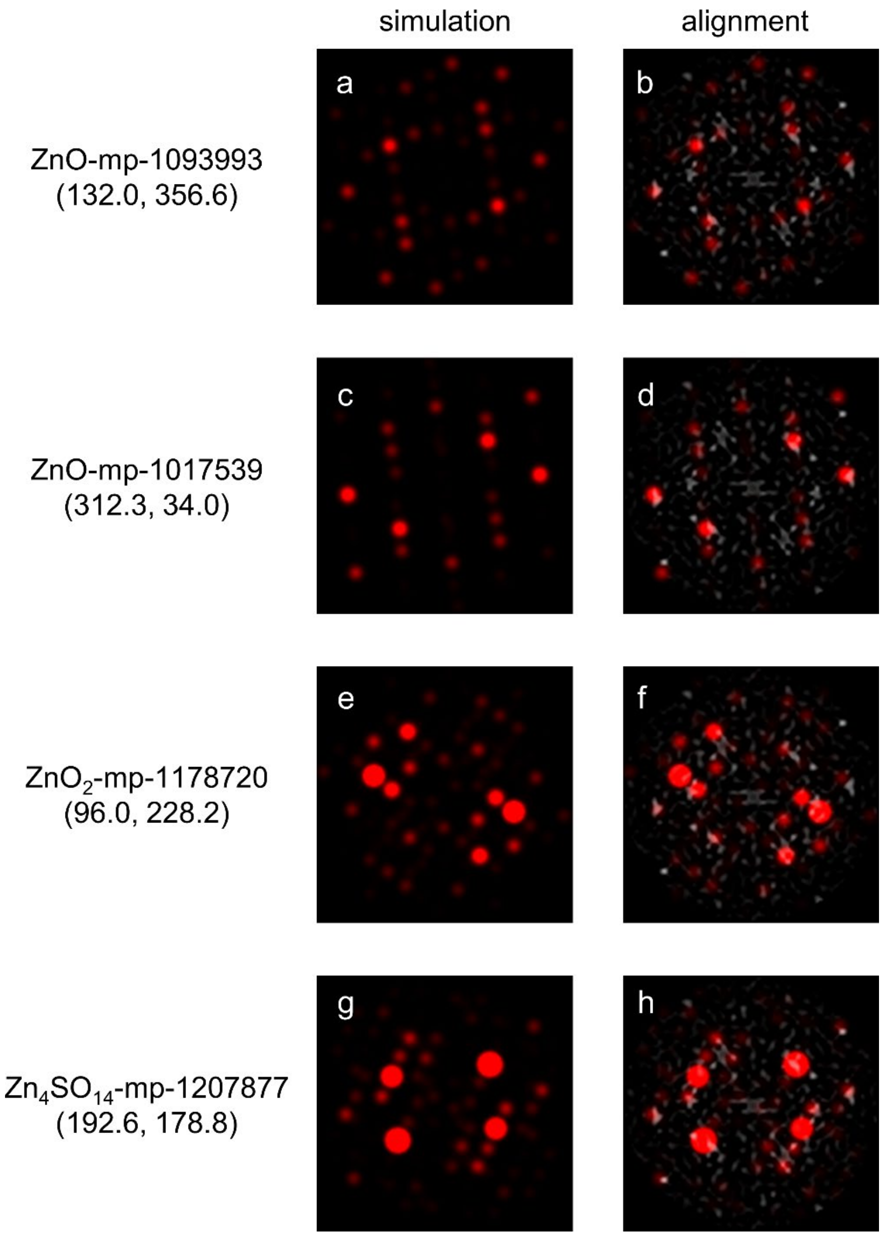

Supplementary Fig. 17. Theoretical reciprocal space of (**a**) ZnO-mp-1093993, (**c**) ZnO-mp-1017539, (**e**) ZnO_2-mp-1178720 and (**g**) Zn_4SO_{14}-mp-1207877. (**b, d, f, h**) The alignment of theoretical vs. actual reciprocal space corresponding to area 3 labeled in Supplementary Fig. 16.

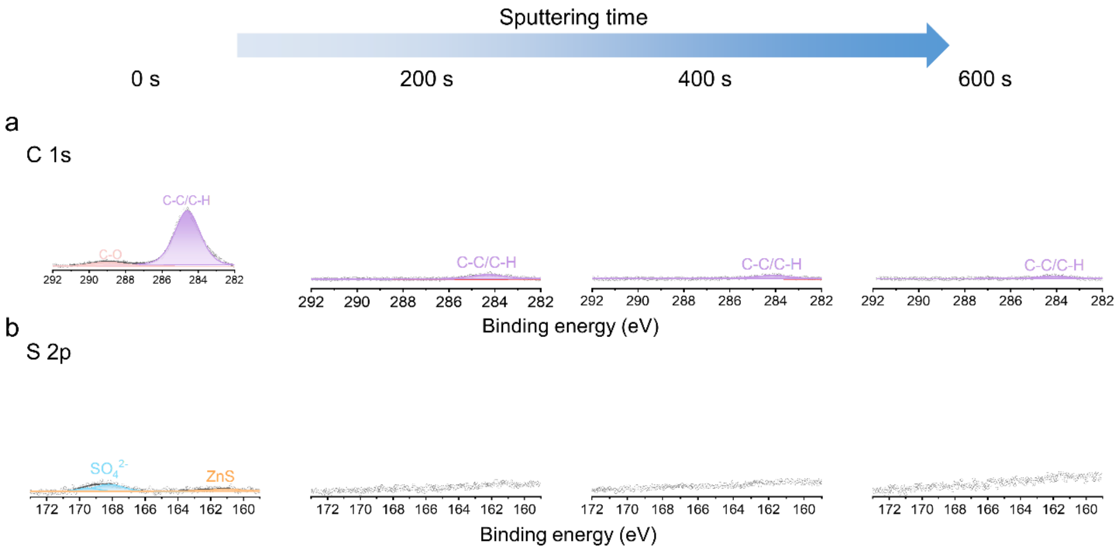

Supplementary Fig. 18. XPS spectra of Zn foil cycling in $ZnSO_4$ electrolyte. (**a**) C 1s and (**b**) S 2p after Ar^+ sputtering from 0 s to 600 s.

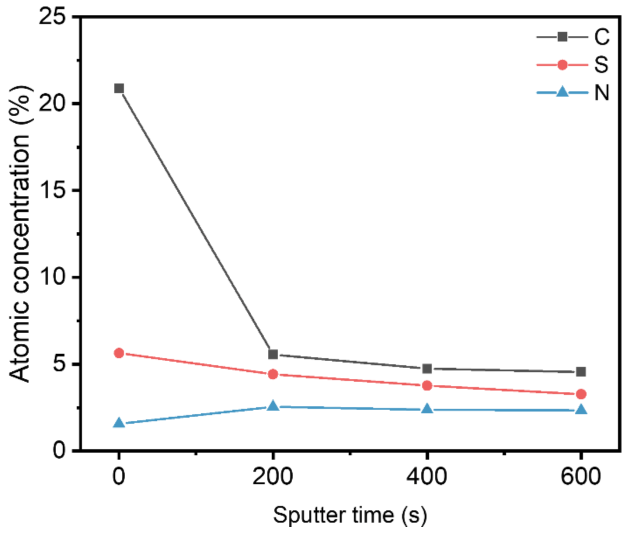

Supplementary Fig. 19. Atomic concentrations of C, N, and S during the XPS Ar^{+} sputtering process.

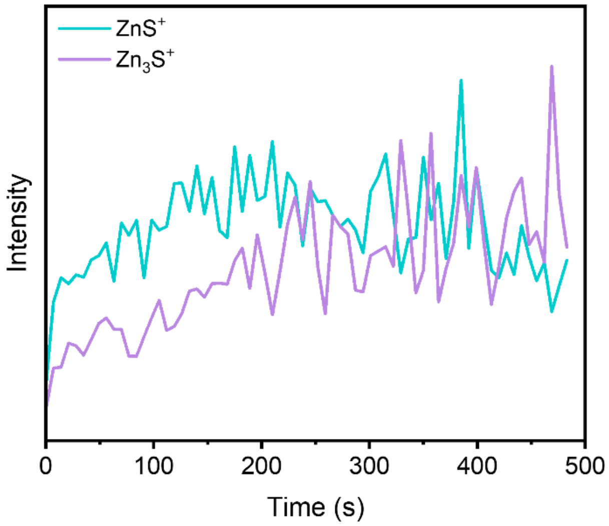

Supplementary Fig. 20. Normalized intensity of the signal of Zn sulfide (Zn_xS_y) in SEI by ToF-SIMS.

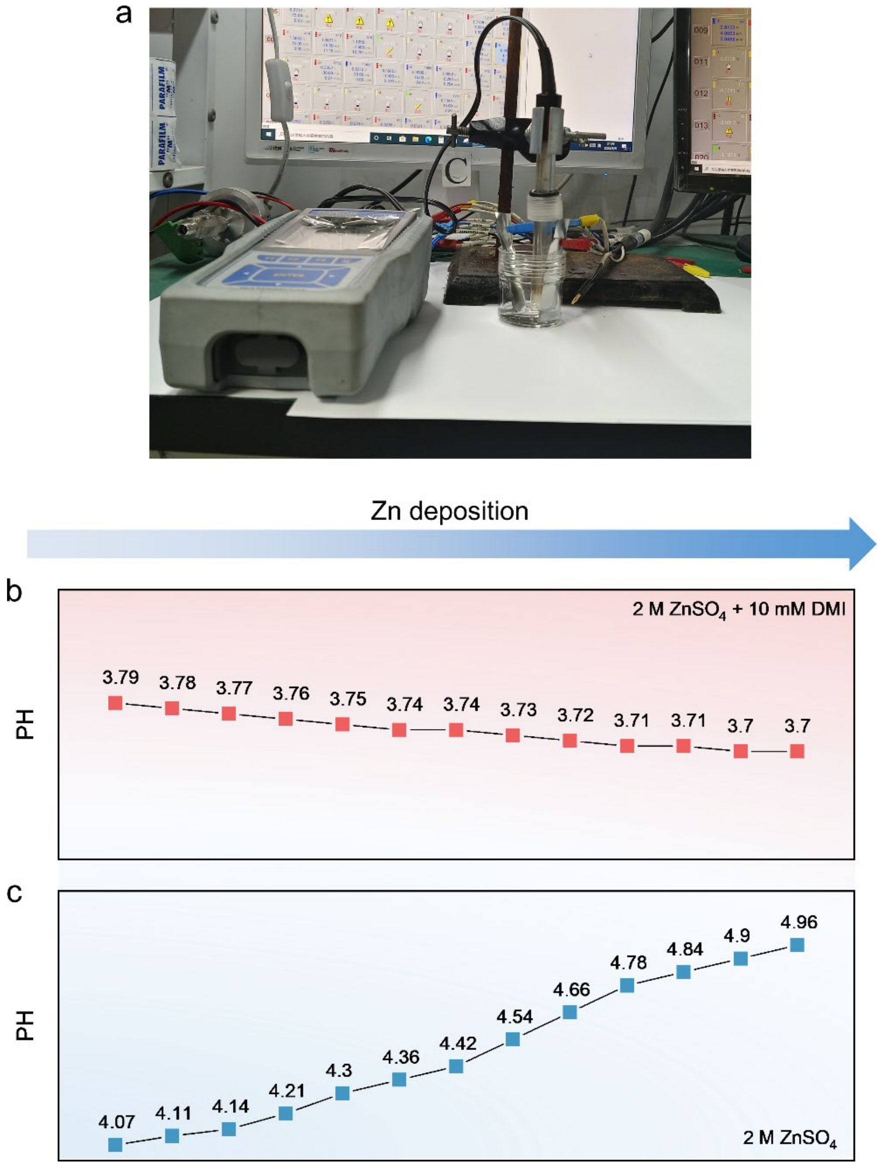

Supplementary Fig. 21. The results of in-situ pH tests during Zn deposition for the first cycle. a, The digital photograph of in-situ pH detection device. **b**, Representative trend profiles of pH changes during Zn deposition in 2 M $ZnSO_4$ + 10 mM DMI electrolyte. c, Representative trend profiles of pH changes during Zn deposition in 2 M $ZnSO_4$ electrolyte.

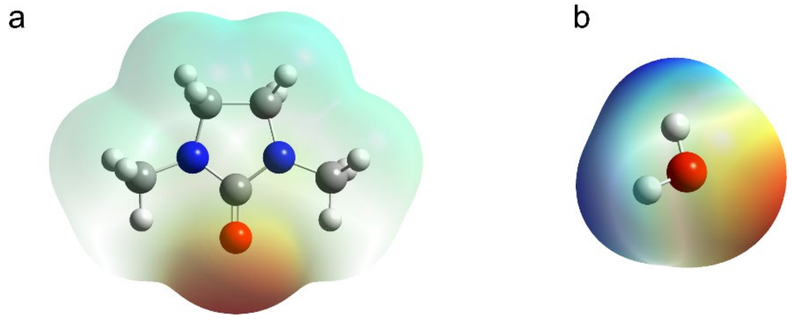

Supplementary Fig. 22. Electrostatic potential mappings of (**a**) DMI and (**b**) H_2O.

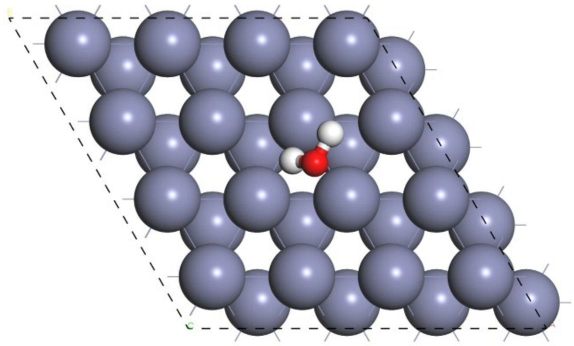

Supplementary Fig. 23. The structure of H_2O adsorbed on Zn surface from the top view.

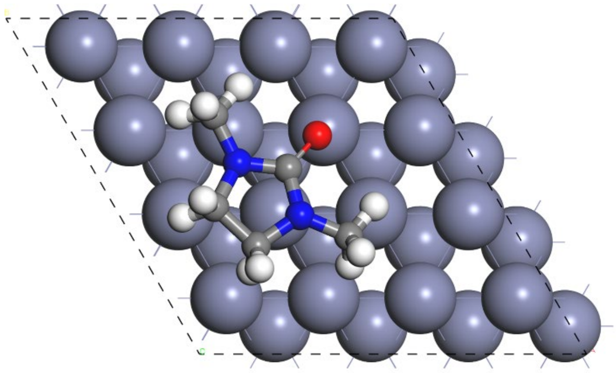

Supplementary Fig. 24. The structure of DMI adsorbed on Zn surface from the top view.

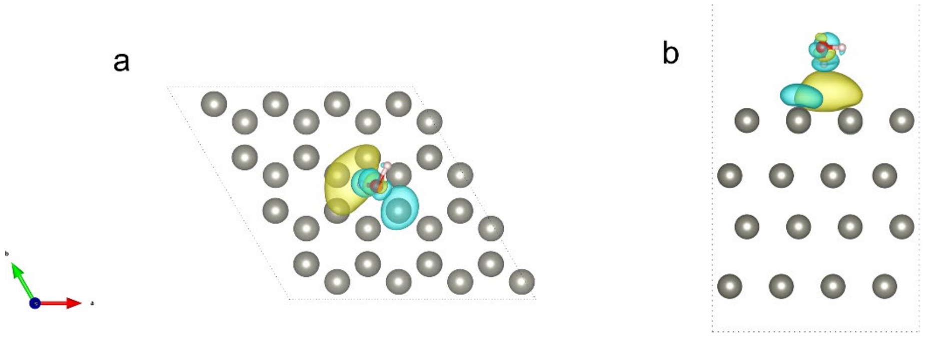

Supplementary Fig. 25. Differential charge density maps of H_2O adsorbed on Zn surface from the (**a**) top view and (**b**) side view.

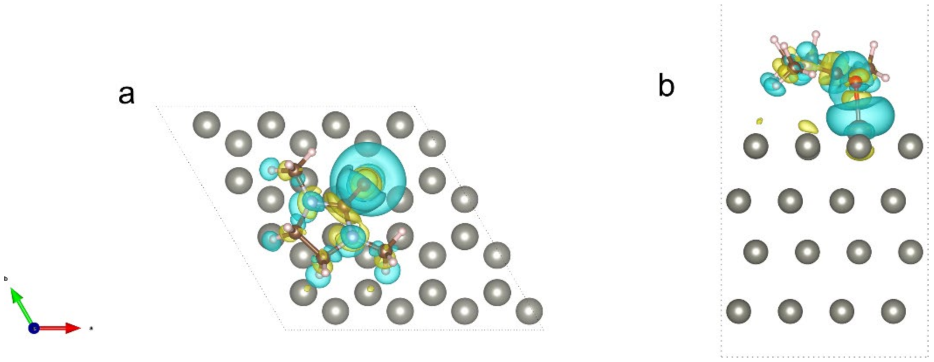

Supplementary Fig. 26. Differential charge density maps of DMI adsorbed on Zn surface from the (**a**) top view and (**b**) side view.

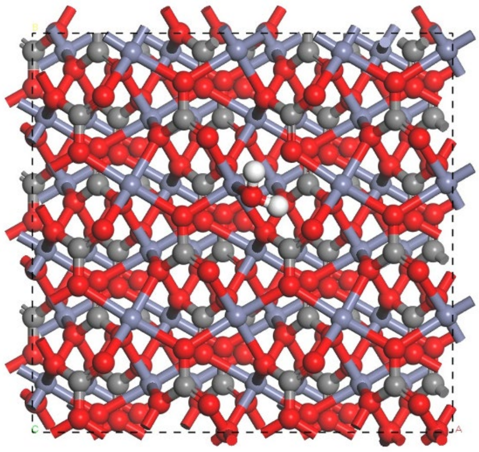

Supplementary Fig. 27. The structure of H_2O adsorbed on $ZnCO_3$ surface from the top view.

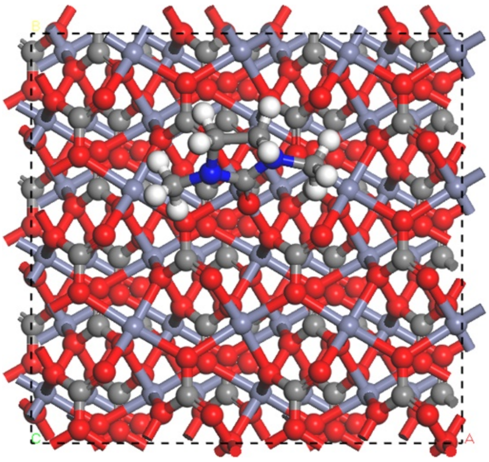

Supplementary Fig. 28. The structure of DMI adsorbed on $ZnCO_3$ surface from the top view.

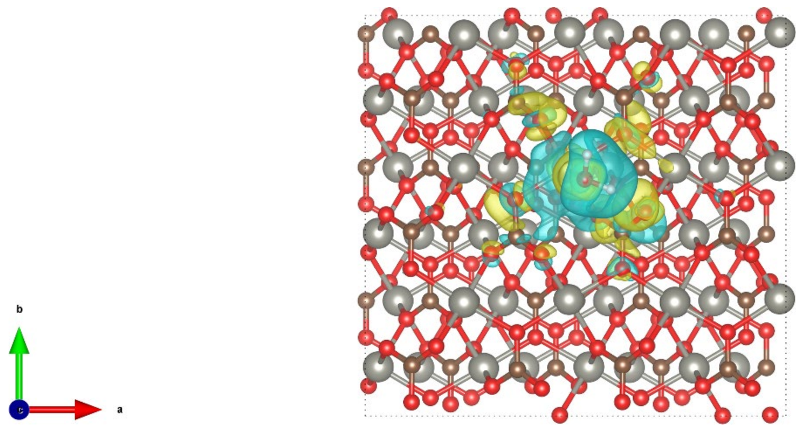

Supplementary Fig. 29. Differential charge density maps of H_2O adsorbed on $ZnCO_3$ surface from the top view.

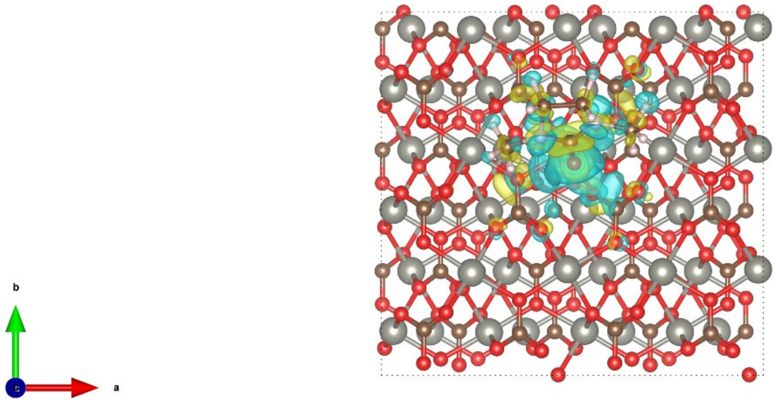

Supplementary Fig. 30. Differential charge density maps of DMI adsorbed on $ZnCO_3$ surface from the top view.

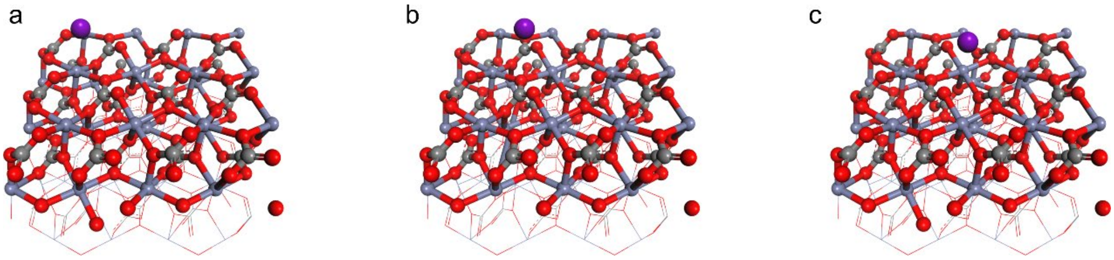

Supplementary Fig. 31. The structures of a Zn atom transport on $ZnCO_3$ in the (a) initial state, (b) transition state, and (c) final state.

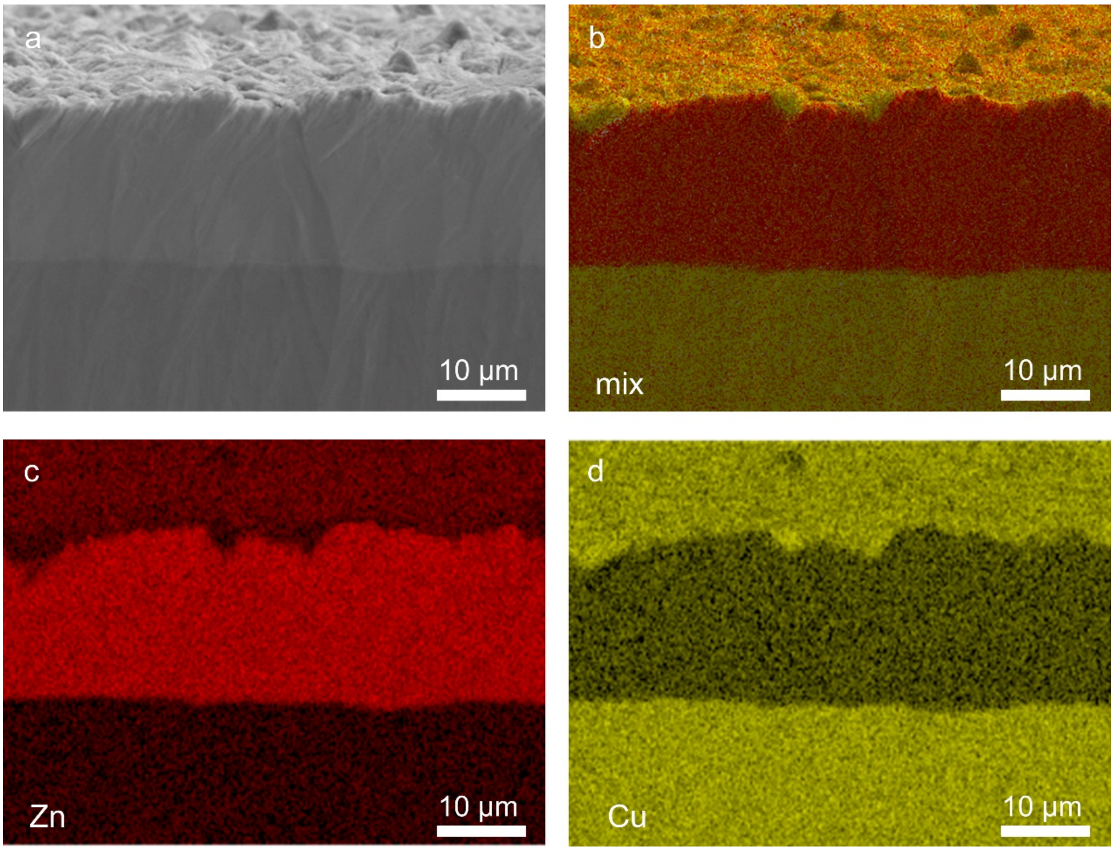

Supplementary Fig. 32. a, Cross-sectional SEM image of Zn deposited on Cu foil with bilayer SEI at 10 mAh cm^{-2}. **b-d**, EDS mapping of (**a**). The slight displacement distribution of Zn and Cu elements is attributed to the sputtering effect during the preparation of cross-sectional sample using ion beam cutting.

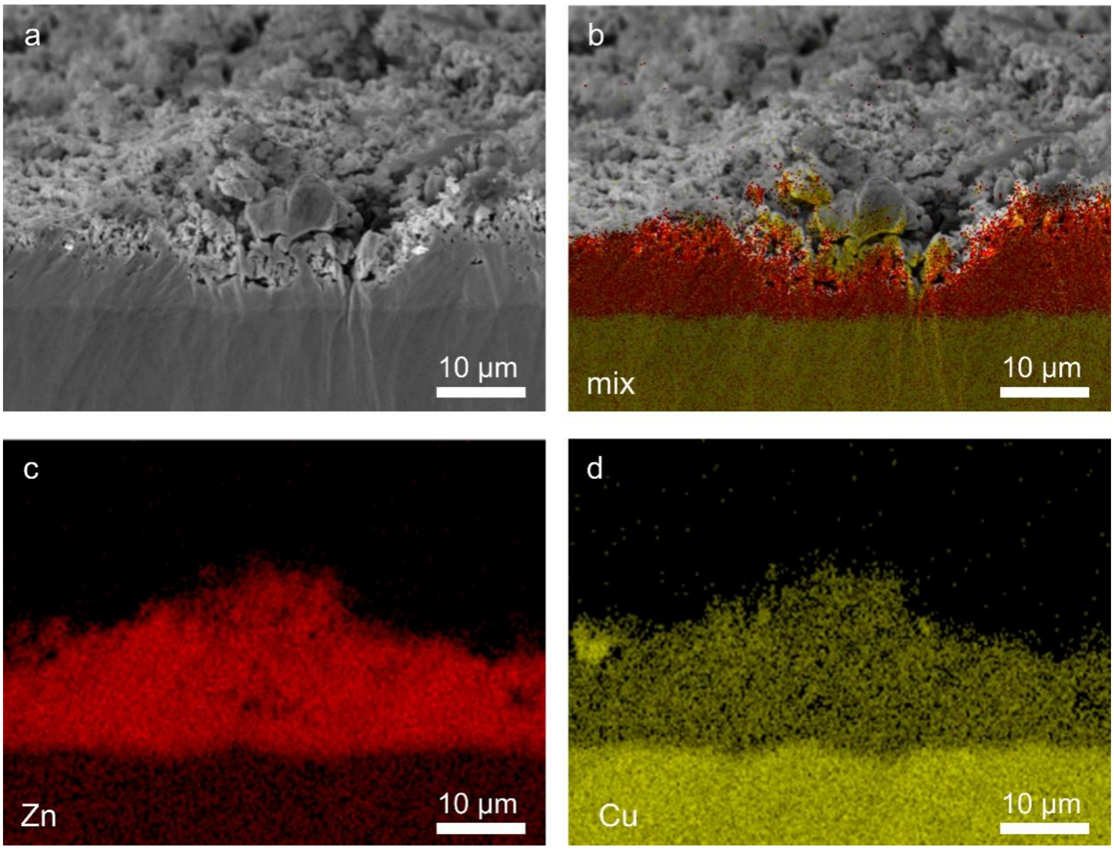

Supplementary Fig. 33. a, Cross-sectional SEM image of Zn deposited on Cu foil without SEI at 10 mAh cm^{-2}. **b-d**, EDS mapping of (**a**). The slight displacement distribution of Zn and Cu elements is attributed to the sputtering effect during the preparation of cross-sectional sample using ion beam cutting.

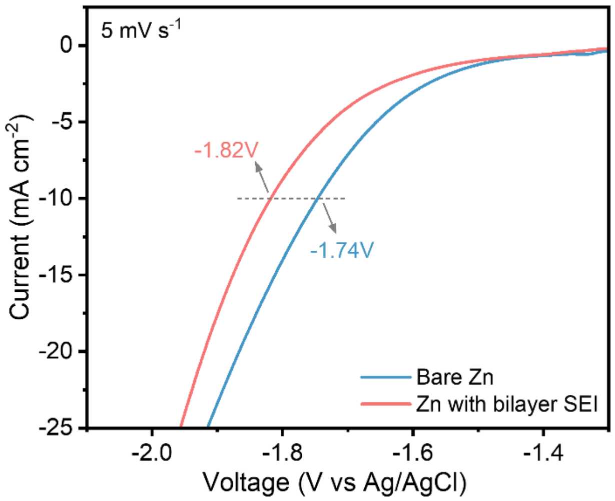

Supplementary Fig. 34. LSV curves of bare Zn or Zn foil with bilayer SEI in 2 M Na_2SO_4 electrolyte at 5 mV s^{-1} (Pt as counter electrode) (25 °C).

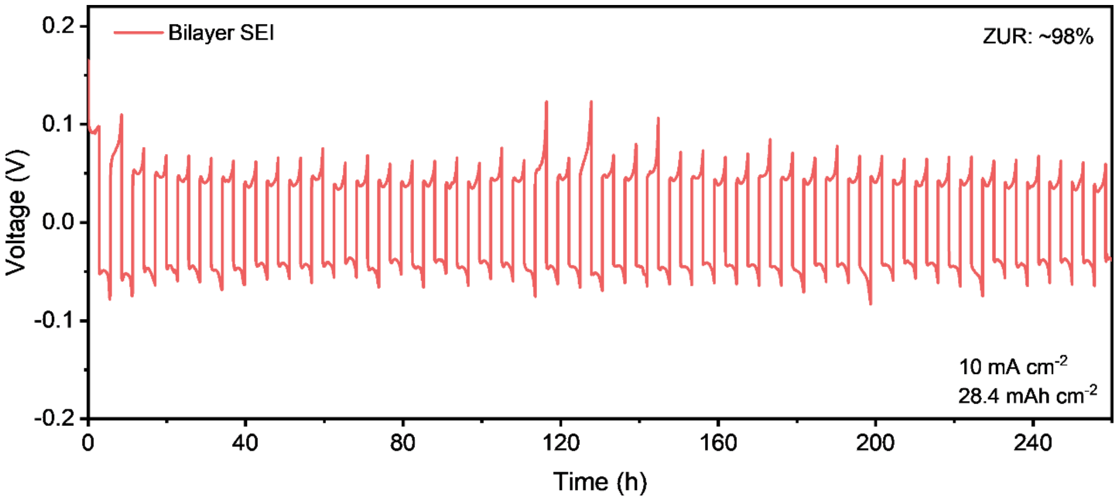

Supplementary Fig. 35. Long-term stability of high ZUR symmetrical cells at 28.4 mAh cm^{-2} and 10 mA cm^{-2} (25 °C).

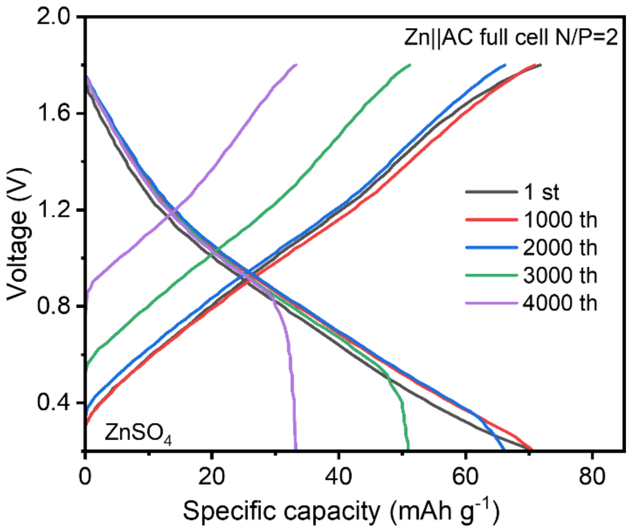

Supplementary Fig. 36. GCD curves of Zn||AC full cell without SEI at 1 A g^{-1} with an N/P ratio of 2 (25 ℃).

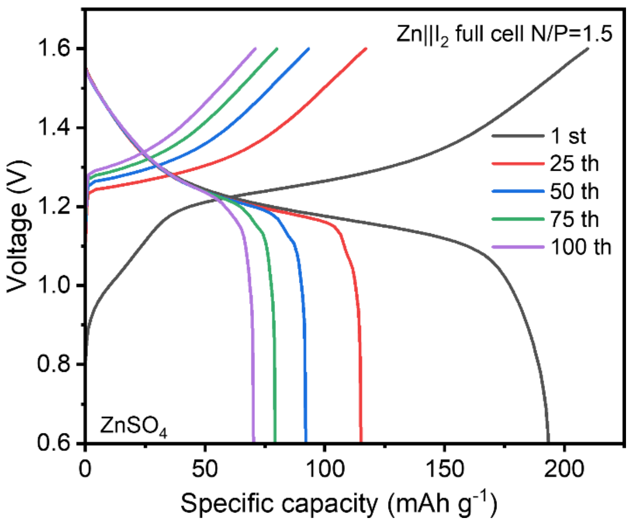

Supplementary Fig. 37. GCD curves of Zn||I_2 full cell without SEI at 2.1 A g^{-1} with an N/P ratio of 1.5 (25 °C).

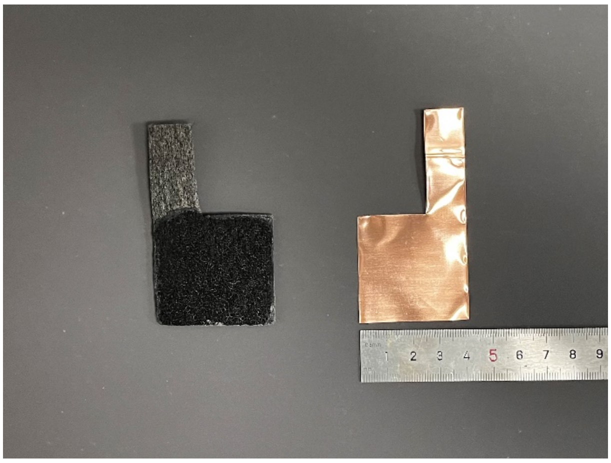

Supplementary Fig. 38. A digital photo of Br positive electrode and Zn-free negative electrode for the Zn||Br pouch cell.

Supplementary Table 1. The energy of reactants and products in the overall formation reaction.

Reactants/Products	Energy (eV)
H_2	-6.811
CO_2	-23.252
H_2O	-14.221
DMI	-102.477
H_2SO_4	-38.188
ZnS	-2.674
$C_4H_{12}N_2$	-96.516
$ZnCO_3$	-28.190
$C_4H_{14}N_2SO_4$	-131.074
$ZnSO_4$	-29.543

Supplementary Table 2. Summary of the electrochemical performance for Zn symmetric cells.

Reference	ZUR	Areal capacity	Lifetime
**This work**	98%	28.4 mAh cm^{-2}	550 h
[1]	96%	24 mAh cm^{-2}	110 h
[2]	90%	9 mAh cm^{-2}	200 h
[3]	90%	11.6 mAh cm^{-2}	420 h
[4]	85.6%	10 mAh cm^{-2}	200 h
[5]	85%	6.5 mAh cm^{-2}	100 h
[6]	80%	5.93 mAh cm^{-2}	160 h
[7]	80%	6.5 mAh cm^{-2}	250 h
[8]	75.5%	13.35 mAh cm^{-2}	300 h
[9]	68%	10 mAh cm^{-2}	100 h
[10]	66.7%	4 mAh cm^{-2}	180 h
[11]	60%	10 mAh cm^{-2}	250 h
[12]	37.5%	10 mAh cm^{-2}	190 h

References

1. Wang, M. et al. High-Capacity Zinc Anode with 96% Utilization Rate Enabled by Solvation Structure Design. *Angew. Chem. Int. Ed.* **62**, e202214966 (2022).
2. Zhang, L. et al. Molecular engineering of self-assembled monolayers for highly utilized Zn anodes. *eScience*, 100205 (2023).
3. Feng, D., Jiao, Y. & Wu, P. Guiding Zn Uniform Deposition with Polymer Additives for Long-lasting and Highly Utilized Zn Metal Anodes. *Angew. Chem. Int. Ed.* **62**, e202314456 (2023).
4. Wang, D. et al. Solvation Modulation Enhances Anion-Derived Solid Electrolyte Interphase for Deep Cycling of Aqueous Zinc Metal Batteries. *Angew. Chem. Int. Ed.* **62**, e202310290 (2023).
5. Yang, X., Zhang, Z., Wu, M., Guo, Z.P. & Zheng, Z.J. Reshaping Zinc Plating/Stripping Behavior by Interfacial Water Bonding for High-Utilization-Rate Zinc Batteries. *Adv. Mater.* **35**, 202303550 (2023).
6. Zhao, R. et al. Lanthanum nitrate as aqueous electrolyte additive for favourable zinc metal electrodeposition. *Nat. Commun.* **13**, 3252 (2022).
7. Li, G. et al. A biocompatible electrolyte enables highly reversible Zn anode for zinc ion battery. *Nat. Commun.* **14**, 6526 (2023).
8. Zhou, K., Li, Z., Qiu, X., Yu, Z. & Wang, Y. Boosting Zn Anode Utilization by Trace Iodine Ions in Organic-Water Hybrid Electrolytes through Formation of Anion-rich Adsorbing Layers. *Angew. Chem. Int. Ed.* **62**, e202309594 (2023).
9. Ming, F. et al. Co-Solvent Electrolyte Engineering for Stable Anode-Free Zinc Metal Batteries. *J. Am. Chem. Soc.* **144**, 7160-7170 (2022).
10. Wang, X. et al. Sandwich-structured anode enables high stability and enhanced zinc utilization for aqueous Zn-ion batteries. *Energy Storage Mater.* **64**, 103078 (2024).
11. Wang, D. et al. Rational Screening of Artificial Solid Electrolyte Interphases on Zn for Ultrahigh-Rate and Long-Life Aqueous Batteries. *Adv. Mater.* **35**, e2207908 (2023).
12. Yuan, W. et al. Realizing Textured Zinc Metal Anodes through Regulating Electrodeposition Current for Aqueous Zinc Batteries. *Angew. Chem. Int. Ed.* **62**, e202218386 (2023).
