# Supplementary material for: Robust bilayer solid electrolyte interphase for Zn electrode with high utilization and efficiency
Source: Nat Commun. 2024 Sep 29;15:8431. doi: 10.1038/s41467-024-52611-z (PMC11439932; doi:10.1038/s41467-024-52611-z)
Supplement: Supplementary file 3 — Description of Additional Supplementary Files [file 41467_2024_52611_MOESM3_ESM.pdf]

Description of Additional Supplementary Files

File Name: Supplementary Data 1

Description: The optimized computational models reported in the manuscript.

File Name: Supplementary Movie 1

Description: The video of in-situ pH tests during Zn deposition for the first cycle in 2 M $ZnSO_4$ + 10 mM DMI electrolyte.

File Name: Supplementary Movie 2

Description: The video of in-situ pH tests during Zn deposition for the first cycle in 2 M $ZnSO_4$ electrolyte.
